# A Comparison of the Learning Effects of Face-to-Face Versus Online Formats of a Clinical Reasoning Lecture

**DOI:** 10.7759/cureus.26109

**Published:** 2022-06-20

**Authors:** Tsuneaki Kenzaka, Ken Goda, Ayako Kumabe

**Affiliations:** 1 Department of Internal Medicine, Hyogo Prefectural Tamba Medical Center, Tanba, JPN; 2 Division of Community Medicine and Career Development, Kobe University Graduate School of Medicine, Kobe, JPN

**Keywords:** rural area, clinical reasoning, face-to-face lecture, in-person lecture, video conference, online lecture

## Abstract

Objective

This study investigated the different learning effects achieved through a clinical reasoning lecture that was simultaneously conducted via two formats: one format involved in-person face-to-face instruction, whereas the other provided remotely conducted online instruction. The lecture was based on a case presentation held at a participating university in 2015.

Methods

We compared the learning outcomes between the abovementioned formats based on participants’ responses. Data were obtained using questionnaires distributed to eligible lecture attendees, including medical students and physicians who had graduated during the preceding 10 years. The questions were about the following aspects: the time duration of the lecture, degree of satisfaction with the online system (online attendees), and degree of satisfaction with the lecture content. The participants then completed a five-question mini-test related to the disease from the presented lecture case to assess their overall degree of understanding.

Results

Online participants gave significantly higher scores for images in the online system (degrees of satisfaction: online 72.7 ± 18.0 vs. in-person 55.6 ± 24.9), audio in the online system (66.1 ± 20.5 vs. 57.5 ± 25.8), the usefulness of multiple venues (82.1 ± 19.3 vs. 60.5 ± 25.0), intention to attend the next lecture (82.3 ± 19.0 vs. 65.8 ± 21.4), and overall meaningfulness of the lecture in the online interactive format (83.6 ± 16.3 vs. 72.6 ± 19.6) compared with the in-person group. However, similar mini-test scores were documented between the two groups (4.2 ± 0.7 for the in-person group and 4.3 ± 0.7 for the online group; no significant differences were noted).

Conclusions

The results show similar learning effects, degrees of satisfaction, and degrees of comprehension between online and in-person lecture attendees. Our findings demonstrate that the online format is a suitable pedagogical intervention in the study context. Continued implementation and further studies are thus warranted to gain deeper insights into the topic.

## Introduction

The traditional pedagogical approach in medical education has focused on face-to-face classroom lectures delivered as per the teacher-centered model [1]. Over the past few decades, this practice has gradually shifted toward implementing online, remote, and electronic learning [2]. However, many obstacles deter the widespread development and implementation of online medical education. For example, problems may arise relating to time constraints, poor technical skills, inadequate infrastructure, absence of appropriate institutional strategies, insufficient support, and negative attitudes among instructors and recipients [3]. Although online learning is gradually evolving to address these issues [2,4], conditions related to the coronavirus disease 2019 (COVID-19) pandemic have increased the demand for a quicker transition to online systems [4]. Unlike the structures of planned e-learning programs and those offered through online systems, many medical schools are now using remote methods to expeditiously provide instruction in entire medical curricula [4]. In light of this, it is highly important to assess the learning outcomes of this approach.

To our knowledge, no previous studies have directly compared the learning effects achieved through in-person lectures versus those delivered from remote locations via online systems. This study directly compared the learning effects of a clinical reasoning lecture that was simultaneously delivered in both the in-person and remote online methods in terms of the degree of comprehension, degree of satisfaction with the lecture content, and degree of satisfaction with the online system in general.

## Materials and methods

Study design

This was an analytical observational study that used questionnaires as the main tool to obtain the data. All procedures were approved by the Institutional Review Board of the Hyogo Prefectural Kaibara Hospital (approval number: Kai-Byo number 1215). Participants were informed that their data would be used for research purposes, and that was explicitly stated in the questionnaires. Additionally, we provided oral and poster presentations to facilitate obtaining informed consent from the participants.

Participants

Eligible respondents of the survey included medical students and physicians who had graduated during the preceding 10 years. The medical students were fifth- and sixth-year students who had completed clinical medicine lectures and were undergoing clinical training as student doctors. In addition, student doctors interested in clinical reasoning voluntarily attended this clinical reasoning lecture. This graduation timeframe was selected based on previous studies that had shown that physicians in Japan become independent approximately 10 years after receiving their degrees [5]. In previous studies conducted in Japan, students and physicians are usually regarded as trainees for a period of up to 10 years post-graduation [5]. In the present study, the survey subjects included those up to their 10th year after graduation, because the lecture was a clinical reasoning lecture using the system 2 clinical reasoning procedure, which is an important concept for trainees [6].

Lecture outline

The clinical reasoning lecture was based on a case presentation held at a university in 2015. The lecture duration was 60 minutes. Attendees included physicians, medical students, and other healthcare practitioners. Lecture participation was voluntary. The case presenter used slides to present a case of intestinal tuberculosis that required a clinical reasoning approach [6], as it had a relatively rare clinical course that could not be immediately diagnosed. In this regard, participants had to think both analytically and systematically while comparing and contrasting illness scripts based on clinical and historical data, demographic characteristics, comorbidities, and epidemiological information [6]. The lecture was conducted in the interactive mode and the lecturer/case presenter asked the participants for their opinions approximately midway through the case presentation and provided explanations [7]. The lecturer used a whiteboard to write down a summary of related cases, differential diagnoses, and the process of clinical reasoning. Finally, an explanation of the disease was provided.

There were two groups: those who attended the lecture in person and those who remotely viewed the presentation using an online system (i.e., the Sony® Television Conferencing System IPELA PCS-XG100®; Sony, Tokyo, Japan). The lecture venue, presentation slides, and whiteboard data were broadcast online to 11 remote medical institutions (A-K) in the Hyogo Prefecture. Except for institution A, all the other medical institutions were located in rural areas. As mentioned, all broadcast elements were simultaneously provided to in-person attendees at the lecture hall. The lecturer/case presenter then held a real-time question-and-answer session with all in-person and online attendees.

Survey implementation

Eligible participants were given questionnaires to complete immediately after the lecture.

Study questionnaire

Information regarding the following aspects was collected from the participants: attendance type (in-person or remote), sex, age, occupation (physician, medical student, or other healthcare practitioners), and either the number of years since graduation (physicians only) or current school year (students only).

Information was collected through a psychometric response scale, that is, visual analog scale (VAS) (range: 0-100 mm or range: -50 to 50 mm); the items are depicted in Table 1. All broadcast elements were simultaneously provided to in-person attendees at the lecture hall. In-person attendance groups rated their level of satisfaction with the images and sounds of the online system through these broadcast elements, rather than the images and sounds physically experienced through direct attendance.

**Table 1:** Questions in the visual analog scale The question concerning the length of lectures used visual analog scale (VAS) values ranging from -50 to 50, with 0 representing just right (i.e., totally satisfactory). VAS values were 0–100 for other questions

Questions
・Concerning the time duration of the lecture
How was the length of the lecture? (long = -50 mm; appropriate = 0 mm; short = 50 mm)
・Concerning the degree of satisfaction with the online system
Were you satisfied with the screen image? (extremely dissatisfied = 0 mm; extremely satisfied = 100 mm)
Were you satisfied with the audio? (extremely dissatisfied = 0 mm; extremely satisfied = 100 mm)
・Concerning the degree of satisfaction with the content of the lecture
Was the content of the lecture educational? (not at all educational = 0 mm; extremely educational = 100 mm)
Was it useful to have an interactive lecture with multiple venues? (not useful at all = 0 mm; extremely useful = 100 mm)
Would you like to attend the next lecture? (not at all = 0 mm; very much so = 100 mm)
Overall, was this lecture meaningful? (not at all = 0 mm; extremely = 100 mm)

Immediately after the lecture, the attendees participated in a mini-test related to the disease discussed during the case presentation. This was done to assess the overall understanding of the lecture content. The test consisted of five questions, each having five options. The response options to the questions and the correct answers are shown in Table 2. The participants were asked to choose the correct answer from the five options.

**Table 2:** Questions, choices, and correct answers for mini-tests related to conference content

Mini-test	
Question 1: which of the following is closest to the tuberculosis incidence rate (number of people per 100,000 population) in Japan in 2014? Choose one:	
(a) 5 people (b) 10 people (c) 15 people (d) 20 people (e) 25 people	Correct answer (c)
Question 2: which of the following is the highest relative risk factor for developing tuberculosis? choose one:	
(a) Hemodialysis (b) Congestive heart failure (c) Gastric ulcer (d) Diabetes mellitus (e) Colon cancer	Correct answer (a)
Question 3: what is the most common site of intestinal tuberculosis? choose one:	
(a) Esophagus (b) Stomach (c) Duodenum (d) Jejunum (e) Ileum	Correct answer (e)
Question 4: which of the following is NOT an anti-tuberculosis drug to be used as a first choice in non-resistant Mycobacterium tuberculosis? choose one:	
(a) Isoniazid (b) Rifampicin (c) Pyrazinamide (d) Ethambutol (e) Bedaquiline	Correct answer (e)
Question 5: which of the following is the recommended response in dealing with a patient with pulmonary TB: (i) mask-wearing by the health care provider - (ii) mask-wearing by the patient? choose one:	
(a) (i) N95 mask - (ii) N95 mask (b) (i) N95 mask - (ii) Surgical mask (c) (i) Surgical mask - (ii) N95 mask (d) (i) Surgical mask - (ii) Surgical mask (e) (i) Surgical mask - (ii) No mask worn	Correct answer (b)

Data analysis

Participants’ attributes and responses were tabulated according to their answers to the questions. The results of the two groups (in-person vs. online) were then comparatively analyzed. Intergroup attribute differences were assessed using the chi-square test, and a t-test was used to compare intergroup responses about the lecture duration, satisfaction with the online system, satisfaction with the lecture content, and lecture comprehension based on mini-test scores. All analyses were conducted using IBM SPSS Statistics for Windows, Version 25.0 (IBM Corp., Armonk, NY); a p-value <0.05 was considered statistically significant.

## Results

Of the 124 total participants, 82 completed the questionnaires (response rate of 66.1%), including 34 (41.4%) in-person attendees and 48 (58.5%) online attendees. The online respondents were from 10 of the 11 remote locations: nine attendees from institution A, 11 from institution B, eight from institution C, two from institution D, zero from institution E (no questionnaires collected), one from institution F, three from institution G, three from institution H, three from institution I, seven from institution J, and one from institution K. All institutions except A were located in rural areas.

Respondents’ ages were as follows: 16 were 20-24 years old (19.5%), 29 were 25-29 years old (35.4%), 14 were 30-34 years old (17.1%), four were 35-39 years old (4.9%), four were 45-49 years old (4.9%), six were 50 years or older (7.3%), and nine did not respond to the question (11.0%). Respondents’ occupations were as follows: 55 physicians (67.1%), 19 medical students (23.2%), six healthcare practitioners (7.3%), and two unknown (2.4%).

Eligible participants who were medical students and physicians who had graduated 10 years prior were selected for the analysis. Hence, 58 eligible participants finally participated in the study, whose details are provided in Table 3. In previous studies from Japan, students and physicians up to 10 years post-graduation are usually regarded as trainees [5]. In the present study, the subjects of the survey included those up to the 10th year after graduation, because the lecture was a clinical reasoning lecture using the system 2 clinical reasoning procedure, which is an important concept for trainees [6]. Ten participants were excluded from the in-person group and 14 from the online group.

**Table 3:** Participants’ characteristics ^†^Significance level: p≤0.05 SD: standard deviation

Characteristics	In-person attendance group (n = 25)	Online attendance group (n = 33)	P-value
Age, years, mean ± SD	25.3 ± 3.6	28.3 ± 3.0	0.001^†^
Males, n (%)	18 (72.0%)	21 (63.6%)	0.352
Age group, n (%)			0.001^†^
20–24 years	13 (52.0%)	3 (9.1%)	
25–29 years	9 (36.0%)	20 (60.6%)	
30–34 years	3 (12.0%)	10 (30.3%)	
Occupation, n (%)			0.001^†^
Medical students, 4th–6th years	18 (72.0%)	0	
Junior residents	2 (8.0%)	19 (57.6%)	
Physicians 3–5 years after graduation	3 (12.0%)	7 (21.2%)	
Physicians 6–10 years after graduation	2 (8.0%)	7 (21.2%)	

The mean age of the in-person group was 25.3 ± 3.6 years and that of the online group was 28.3 ± 3.0 years (significantly higher in the online group). Regarding occupation, the in-person lecture group mainly comprised medical students in their fourth to sixth years of study, whereas the online lecture group comprised junior residents and physicians. However, there were no significant intergroup differences with regard to gender. Table 4 shows the results of each item rated using VAS.

**Table 4:** Results for each question in the visual analog scale *Significance level: p≤0.05 The question concerning the length of lectures used visual analog scale (VAS) values ranging from -50 to 50, with 0 representing just right (i.e., totally satisfactory). VAS values were 0–100 for other questions SD: standard deviation. Difference (post−pre): difference in scores before and after the program. CI: confidence interval

Details of questions	(A) In-person lecture group, mean ± SD	(B) Online lecture group, mean ± SD	Difference (A-B) (95% CI)	P-value
・Concerning the time duration of the lecture				
How was the length of the lecture?	7.0 ± 18.0	4.7 ± 12.2	2.3 (‐6.2 – 10.8)	0.585
・Concerning the degree of satisfaction with the online system				
Were you satisfied with the screen image?	55.6 ± 24.9	72.7 ± 18.0	‐17.1 (‐28.6 – ‐5.6)	0.004*
Were you satisfied with the audio?	57.5 ± 25.8	66.1 ± 20.5	‐8.6 (‐18.5 – ‐2.4)	0.039*
・Concerning the degree of satisfaction with the content of the lecture				
Was the content of the lecture educational?	80.9 ± 12.6	82.0 ± 17.1	‐1.1 (‐9.3 – 7.1)	0.785
Was it useful to have an interactive lecture with multiple venues?	60.5 ± 25.0	82.1 ± 19.3	‐21.6 (‐33.8 – ‐9.3)	0.001*
Would you like to attend the next lecture?	65.8 ± 21.4	82.3 ± 19.0	‐16.5 (‐27.3 – ‐5.7)	0.003*
Overall, was this lecture meaningful?	72.6 ± 19.6	83.6 ± 16.3	‐11.0 (‐20.6 – ‐1.5)	0.024*

Participants in both groups reported that the lecture duration (60 minutes) was appropriate, with no significant differences between the groups. Regarding the online system, remote participants were generally satisfied with the screen image (72.7 ± 18.0) but indicated that the audio could be improved (66.1 ± 20.5). The degrees of satisfaction with the screen image and audio were significantly higher among the online group when compared with the in-person group. Regarding the lecture content, VAS values for the following items were significantly higher among the online group when compared with the in-person group:

“Was it useful to have an interactive lecture with multiple venues?”

“Would you like to attend the next lecture?”

“Overall, was this lecture meaningful?”

Regarding the degree to which participants understood the lecture content, the mean numbers of correct responses to the mini-test were 4.2 ± 0.7 for the in-person group and 4.3 ± 0.7 for the online group (no significant intergroup differences) (Table 5).

**Table 5:** Degree to which the respondents understood the lecture content SD: standard deviation. Difference (A-B): differences in the (A) in-person lecture group and (B) online lecture group. CI: confidence interval

	(A) In-person lecture group, mean ± SD	(B) Online lecture group, mean ± SD	Difference (A−B) (95% CI)	P-value
Number of correct responses to 5 questions	4.2 ± 0.7	4.3 ± 0.7	‐0.1 (‐0.5 – 0.2)	0.748

## Discussion

This study investigated a clinical reasoning lecture that was simultaneously delivered using an online and in-person method to determine the learning effects in terms of the degree of comprehension, degree of satisfaction with the lecture content, and degree of satisfaction with the online system (remote participants only).

Regarding the degree to which participants understood the lecture content, the numbers of correct responses to the five-question mini-test were 4.2 ± 0.7 for the in-person group and 4.3 ± 0.7 for the online group (no significant differences). The mini-test was given immediately after the lecture, and all the questions could be answered if the participants had listened to the lecture appropriately. Therefore, it was not a pure knowledge comparison, but rather a test of whether the participants had paid attention to the lecture content carefully, including the audio and images. Hence, even if there had been a knowledge gap before the lecture, it would have little effect on the results of the post-lecture mini-test, but rather would be related to whether or not the student was able to listen to the lecture carefully and thoroughly. Both groups demonstrated a high degree of understanding; the degree of understanding of the learning content obtained from the lecture rated after the lecture was almost the same. Notably, 72.0% of the in-person group comprised medical students, whereas 57.6% of the online group comprised junior residents. Although there were varying degrees of expertise, most participants were learning about a relatively rare case using the system 2 clinical reasoning procedure for the first time. Clinical reasoning system 2 is used for cases that cannot be immediately diagnosed [6]. System 2 is characterized by thinking analytically and systematically in addition to comparing and contrasting illness scripts in light of emerging data elicited from history and examination while factoring in demographic characteristics, comorbidity, and epidemiologic data [6]. The lecturer/case presenter provided a detailed summary of the presented case, including the illness itself, and assessed the overall degree of understanding accordingly. As such, all mini-test questions could be correctly answered if participants had paid attention during those components. Based on those conditions, neither the level of participant expertise nor their professional status might have influenced the results.

As mentioned earlier, respondents were asked about the usefulness of an interactive lecture with multiple venues, their degree of satisfaction with the lecture content, and whether they would like to attend a subsequent lecture. Regarding whether the lecture was considered meaningful overall using the online interactive format, the online lecture group reported significantly higher scores (83.6 ± 16.3) than the in-person lecture group (72.6 ± 19.6). However, this does not simply indicate that online lectures are more satisfying than in-person lectures; in fact, most online participants worked at medical institutions in rural areas of the Hyogo Prefecture, and the limited access to lectures and study sessions might have influenced the high degree of satisfaction in the online group. Moreover, the practical lecture content on clinical reasoning was based on an actual case, which might have influenced the results since many physicians in the online group were involved in actual clinical practice. Nevertheless, similar degrees of satisfaction were reported by both groups.

Regarding the degree of satisfaction with the online system, the online lecture group recorded a score of 72.7 ± 18.0, indicating general satisfaction. However, the degree of satisfaction with the audio was lower, based on a score of 66.1 ± 20.5, which indicates there was room for improvement. The images broadcast online were physically provided at the lecture hall. In this regard, the in-person group reported a degree of satisfaction of 55.6 ± 24.9 with the screen image, which was much lower than that reported by the online group. However, the lecture was held in a larger hall than usual because of the need to accommodate an appropriately sized screen for online broadcasting, which might have influenced both the audio and screen results for the in-person group.

Previous studies have reported on the key barriers that affect the development and implementation of online medical education, including time constraints, poor technical skills, inadequate infrastructure, absence of institutional strategies, lack of support, and negative attitudes among instructors and recipients [3]. However, research has also shown that digital tools can solve the issue of time constraints [8]. In this study, both groups reported high levels of understanding based on the lecture content, meaning that time- and distance-related constraints could be solved through such implementations. Negative attitudes could likely be resolved by taking sufficient preparatory measures. This is especially important considering the difficulty associated with conducting face-to-face lectures during the COVID-19 pandemic, which has brought about many social distancing rules [9]. Alternative and user-friendly platforms such as Facebook®, Zoom®, and Skype® could also be used in medical education [10], which might help improve negative attitudes among all concerned parties. Infrastructural inadequacies can also be resolved by using appropriate platforms. In summary, many major barriers to education are now being resolved, but some challenges still remain. Interventions should thus be targeted at improved technical skills, sufficient institutional strategies, and increased support.

Limitations

This study had some limitations. Regarding the questionnaire completion rate, all persons who entered the lecture hall or online lecture venues were counted as participants. As it was possible to freely exit the hall, approximately 20 persons (almost equal numbers in the in-person and the online groups) left during the lecture, and they were therefore not exposed to the whole lecture and its content. Nevertheless, 82 of 124 persons having completed the questionnaire can be considered reasonable. Regarding the analyzed participants, the in-person group contained many medical students, whereas the online group had many junior residents. This was probably because the in-person lecture was held at a university, whereas the online lecture was held at various training hospitals. The lecture was about a relatively rare case that required the use of system 2 clinical reasoning. As the lecturer had thoroughly explained all the content, we believe that participant type/affiliation had little effect on the reported degree of understanding. Furthermore, as the lecture was based on clinical reasoning, the interactive format should have facilitated content comprehension. Subjects who had detailed knowledge in advance were considered to be quite rare. However, since there was a statistically significant difference in age and occupation between the two groups and thus possibly experience and medical knowledge, this might have affected the results. The five questions were not asked before the lecture to examine baseline knowledge and then compare results after the lecture to ascertain which group improved more. This constitutes another limitation of this study. Additionally, this study did not investigate how one-way lectures affected the degree of understanding. We addressed this problem during the lecture summary, in which the lecturer verified whether participants comprehended the described disease; this component was conducted in the one-way format. In this regard, we do not believe there were any issues in terms of knowledge provision. Because each individual learns in a different way, one should be cautious about generalizing the results of this study.

## Conclusions

Comparing the learning effects of in-person and real-time online lectures among fourth- to sixth-year medical students and physicians up to 10 years post-graduation, in terms of comprehension, we found that the online format was not inferior to the direct in-person format. Additionally, online participants gave significantly higher scores for images in the online system, audio in the online system, the usefulness of having multiple venues, intention to attend the next lecture, and overall meaningfulness of the lecture using the online and interactive format compared with the in-person group.

## References

[1] (2022). Albarrak A: education in a technological world: communicating current and emerging research and technological efforts 1st ed. Formatex Research Center. http://www.formatex.info/ict/book/147-153.pdf.

[2] Shachar M, Neumann Y (2022). Shachar M, Neumann Y: differences between traditional and distance education academic performances: a meta-analytic approach. The International Review of Research in Open and Distributed Learning. http://www.irrodl.org/index.php/irrodl/article/view/153/704.

[3] O'Doherty D, Dromey M, Lougheed J, Hannigan A, Last J, McGrath D (2018). Barriers and solutions to online learning in medical education - an integrative review. BMC Med Educ.

[4] Camargo CP, Tempski PZ, Busnardo FF, Martins MA, Gemperli R (2020). Online learning and COVID-19: a meta-synthesis analysis. Clinics (Sao Paulo).

[5] Furukawa M, Kumagai Y, Tajima A (1995). An experiment in the introduction to Medicine Course: the self-image of physicians ten years after graduation. Igaku Kyoiku/Med Educ (Japan).

[6] Thampy H, Willert E, Ramani S (2019). Assessing clinical reasoning: targeting the higher levels of the pyramid. J Gen Intern Med.

[7] Watanuki S, Sada R, Ishikane M, Shimizu T, Kutsuna S (2018). The Tokyo GIM Conference: clinical reasoning conference from real cases. J Gen Fam Med.

[8] Dyrbye L, Cumyn A, Day H, Heflin M (2009). A qualitative study of physicians' experiences with online learning in a masters degree program: benefits, challenges, and proposed solutions. Med Teach.

[9] An L, Hawley S, Van Horn ML, Bacon E, Yang P, Resnicow K (2021). Development of a coronavirus social distance attitudes scale. Patient Educ Couns.

[10] Gonzales-Zamora JA, Alave J, De Lima-Corvino DF, Fernandez A (2020). Videoconferences of infectious diseases: an educational tool that transcends borders. A useful tool also for the current COVID-19 pandemic. Infez Med.

